# The new biologic drugs: Which children with asthma should get what?

**DOI:** 10.1002/ppul.27218

**Published:** 2024-09-13

**Authors:** K. Hillson, S. Saglani, A. Bush

**Affiliations:** ^1^ National Heart and Lung Institute, Imperial College London London UK; ^2^ Pediatric Respiratory Medicine, Royal Brompton and Harefield Hospitals London UK

**Keywords:** asthma, biologics, pediatrics, treatment

## Abstract

Novel biologics (targeted antibody therapies) have revolutionized the management of severe childhood asthma. However, it is important that the right biologic is selected for the right patient, and understanding the evidence base for each biologic is crucial. Currently, four biologics (all monoclonal antibodies) are licensed in the UK for the treatment of children with severe asthma ‐ omalizumab (Xolair), mepolizumab (Nucala), and dupilumab (Dupixent) in children aged 6 years and over; and tezepelumab (Tezspire), only in children aged 12 years and over. Tezepelumab is the only licensed biological that may be beneficial in severe asthma without evidence of Type 2 inflammation. All have a good safety profile but varying degrees of clinical efficacy in children, with wide variation in treatment responsiveness between individual patients. When selecting biologics for severe asthma, it is essential to remember the limitations of the current pediatric evidence. At present, there are no results from randomized, head‐to‐head trials of biologics in severe asthma. TREAT is an ongoing trial comparing omalizumab to mepolizumab and will be one of the first to provide such evidence. We must be especially aware of the dangers of extrapolating data from adults to children, because the pathophysiology and role of biomarkers may differ significantly from adult asthma. Given the current level of knowledge, even after treatment has been initiated, children should be regularly reviewed to determine the efficacy of treatment, side‐effect profile and consideration of when treatment with the biologic should be discontinued.

## 1 INTRODUCTION

Asthma affects one in 11 children in the UK, one of the highest prevalences of this condition in Europe.^1^ This disease burden has not significantly changed over the last two decades, and the UK has some of the worst asthma outcomes (including childhood asthma deaths) in Europe.^2^, ^3^, ^4^ Asthma was classically thought to be variable airflow flow obstruction, caused by inflammation and bronchial hyper‐responsiveness. However, more evidence is emerging that asthma is a heterogeneous condition. Management of severe asthma is challenging, particularly since the evidence base for treatment is largely extrapolated from teenagers and adults with severe asthma, which is recognized to have a different pathophysiology to school aged asthma. Evidence has also emerged that children with mild or moderate asthma may have different pathophysiology to those with severe asthma.^5^ It is estimated that around 2%–5% of children with school aged asthma have problematic severe asthma (PSA), with poor symptom control despite being prescribed maximum asthma therapy.^6^ It is crucial to recognize that children with PSA account for half of healthcare costs for childhood asthma and are at high risk of asthma related deaths, and therefore all children with severe asthma must be referred to a severe asthma specialist center.

**Figure 1:**
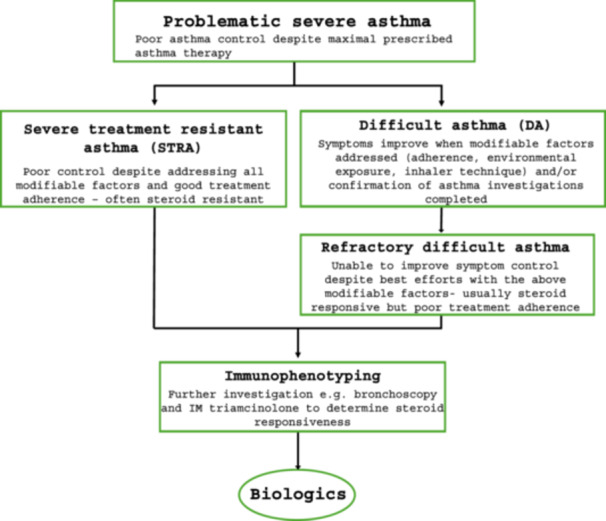
Severe asthma classification and consideration for biologics in children.

Children with PSA can be further divided into two groups—difficult asthma (DA) and severe treatment resistant asthma (STRA) (Figure 1).^7^ Most children with PSA fall under the DA category, and these children benefit from having access to a multidisciplinary specialist asthma team, where modifiable factors can be investigated and addressed. However, a small percentage of children still fall into the “refractory difficult asthma” group, where severity of symptoms is due to noncompliance with treatment, poor inhaler technique despite education, unmodifiable exposure to environmental triggers or medical comorbidities despite maximal efforts to address them.^7^

The European Respiratory Society defines “severe treatment refractory asthma” (STRA) in children as “encompassing a variety of sub‐phenotypes of asthma that do not respond to current standard therapy, that is, high doses of inhaled corticosteroids in combination with long‐acting β2‐agonists (LABA).”^8^ These children have a different immunophenotype compared to the children with DA.^7^ The introduction of novel biologics, which is licenced for use in this group, has revolutionized the management of many of the patients with STRA. When used appropriately, biologics can make a significant difference (especially to asthma attack frequency and severity), improve symptom control, improve quality of life and may allow reduction of other maintenance therapies in children with STRA. However, we also advocate for the use of biologics in children with “refractory difficult asthma,” to safeguard these children from severe asthma attacks.^5^

## 2 BASIC IMMUNOPATHOLOGY OF ASTHMA

Asthma can be broadly subdivided into Th‐2 high or Th‐2 low endotypes. Pediatric asthma predominantly has a Th‐2 high endotype, involving raised levels of blood and airway eosinophils and allergen senitization (the Th‐2 low variant is more common in adults).^9^

**Figure 2:**
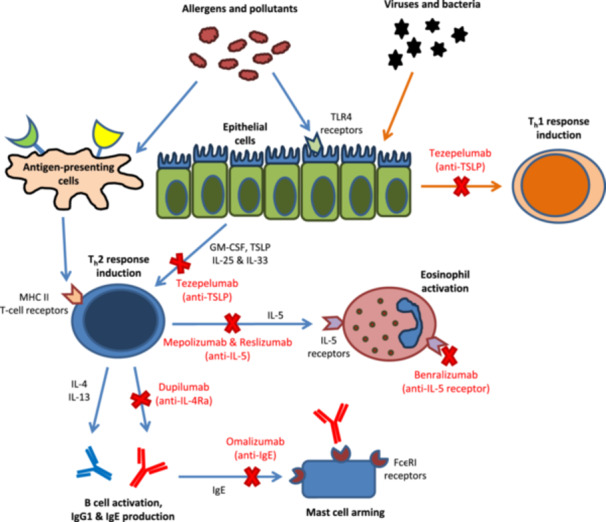
Simplified diagram of Type 1 and Type 2 inflammatory pathways, showing the primary signaling molecules involved in each step and summary of mechanism of action of available biologics for severe asthma. Abbreviations: FC∊RI = high‐affinity IgE receptor; Ig = Immunoglobulin; IL = Interleukin; MHC II = Major histocompatibility complex II; TLR4 = Toll‐like receptor 4; TSLP = thymic stromal lymphopoietin.

Th‐2 high asthma is driven (amongst other stimuli) by inhalation of allergens to which the patient is sensitized. This triggers antigen presenting cells (APCs) to bind to major‐histocompatibility (MHC) T cell receptor II, which activates CD‐4 + T cells and initiates a molecular cascade which results in the secretion of cytokines IL‐4, IL‐5, and IL‐13. IL‐4 switches antibody‐producing B cells to IgE synthesis, which in turn leads to the activation of mast cells and basophils through the binding of IgE to their FC∊RI receptors.^10^ IL‐5 is important in the growth, recruitment, activation, and survival of eosinophils, which contribute to acute asthma attacks as well as activating the pathway that leads to production of IgE. IL‐13 leads to increased mucus production and raised fractional exhaled nitric oxide (FeNO), a biomarker of Th‐2 inflammation. There are also upstream innate immune components to the Th‐2 response, which are cytokines (alarmins) synthesized and released by bronchial epithelial cells (e.g., IL‐33, IL‐25 and thymic stromal lymphopoietin [TSLP]). The recruitment of type 2 innate lymphoid cells (ILC2 cells) by these cytokines leads to an increase in granulocyte‐macrophage colony‐stimulating factor (GM‐CSF), IL‐5, and IL‐13 production. The connections between these signaling molecules and the processes that they control are illustrated in Figure 2 below.

## 3 NOVEL BIOLOGICS FOR SEVERE ASTHMA: MECHANISM OF ACTIONS AND ELIGIBILITY CRITERIA

Currently, six biologics are available in the United Kingdom (UK), on the NHS for the treatment of asthma ‐ omalizumab (Xolair), mepolizumab (Nucala), reslizumab (Cinqaero), benralizumab (Fasenra), dupilumab (Dupixent) and tezepelumab (Tezspire). However, only four—mepolizumab, omalizumab, dupilumab and the newly approved tezepelumab (since April 2023)—have been licenced for use in children with severe asthma in the UK.^11^, ^12^ Before commencing treatment with any biologic, Benralizumab (Fasenra, Astra Zeneca) is currently not licenced in the UK, but has been approved by the FDA for children with severe eosinophilic phenotype asthma in the USA, and is only briefly covered in this review. National Institute for Health and Care Excellence (NICE) guidelines advise that it is crucial to ensure that standard therapy has been optimized, including documenting adherence with maintenance inhaled steroid treatment as far as possible, optimal inhaler technique, and attention to minimizing environmental exposures that may worsen control (allergens, tobacco smoke). However, adherence may be difficult to assess accurately. There is justification to prescribe biologics to children with severe asthma who, despite best efforts to optimize adherence, remain non‐adherent to conventional treatment, to keep them alive. All currently licensed biologics are antibodies which target signaling molecules, summarized in Figure 2.

## 4 OMALIZUMAB

### 4.1 Mechanism of action

Omalizumab (Xolair) is a recombinant humanized monoclonal anti‐IgE antibody that forms complexes with free serum IgE, reducing the amount of free IgE that is available to bind and arm mast cells and basophils via their high affinity FC∊RI receptors, effectively decreasing the inflammatory response to allergen exposure (Figure 2).^13^, ^14^ In turn, this effect also leads to the downregulation of surface expression of mast cell and basophil FC∊RI.^15^ The resultant reduction in functional cross‐linking of FC∊RI reduces symptoms and especially asthma attacks in children with allergic IgE‐mediated asthma.^16^ In addition, there is convincing evidence that omalizumab has antiviral effects. Children with allergic asthma are hypothesized to be more susceptible to viral infections due to high expression of FcεRIα on dendritic cells, and IgE cross‐linking downregulates IFN‐α antiviral responses. Omalizumab may reduce the expression of FcεRIα and thereby augment antiviral responses in these children.^17^

### 4.2 Indications

In the UK, the NICE has approved the use of omalizumab in children aged 6 years and over with confirmed severe, persistent, IgE mediated allergic asthma, who require frequent treatment with oral corticosteroids (defined as four or more courses in the previous year) despite optimized therapy. Patients must have a serum IgE within 30–1500 IU/ml, and evidence of senitization to at least one perennial aeroallergen, regardless of eosinophil count (Table 5).^18^ This need for senitization may be unnecessary, because in adults with asthma and a raised IgE, omalizumab is effective even if there is no detectable aeroallergen senitization.^19^, ^20^ Omalizumab is given subcutaneously every 2–4 weeks and the dose is determined by baseline serum IgE and body weight. The treatment is trialed for 16 weeks and continued if there is evidence of improvement in symptom control, rate of attacks or quality of life, as judged by their clinical team. Table 1 summarizes the main pediatric studies looking at the efficacy of omalizumab.

**Table 1:** Summary of main pediatric studies looking at the efficacy of omalizumab, administered subcutaneously.

Study	Number of participants and age	Major Inclusion criteria	Summary of evidence
Milgrom et al.^21^ Double‐blind, randomized, placebo‐controlled trial.	Three hundred thirty‐four children Omalizumab (*n* = 225) Control (*n* = 109) Age 6–12 years	Moderate to severe allergic asthma requiring treatment with inhaled corticosteroids. Total serum IgE level of 30–1300 IU/ml Well controlled for ≥3 months with ICS (168–420 μg/d BDP equivalent)	More participants in the treatment group achieved sustained reduction of budesonide dose, with greater magnitudes of reductions compared to control (median reduction 100% in omalizumab group vs. 66.7% in control). Complete budesonide withdrawal achieved in 55% (treatment) versus 39% (control) During the steroid reduction phase of treatment: i. proportion of children with asthma attacks was 18.2% (treatment) versus 38.5% (control) ii. Mean number of attacks per patient which required treatment with either doubling of budesonide dose or the administration of systemic corticosteroids was 0.42 (treatment) versus 2.72 (control) Reduction in number of missed school days, unscheduled hospital visits, serum IgE in the treatment group. No significant difference in lung function and asthma symptom score in treatment compared to control group.
Lanier et al.^22^ Kulus et al.^23^ Double blinded, randomized, placebo‐controlled trial	626 children Omalizumab (*n* = 421) Placebo (*n* = 206) Age 6–12 years	Moderate‐to‐severe allergic (IgE‐mediated) asthma with evidence of perennial allergen sensitivity. Total serum IgE level 30–1300 IU/ml Uncontrolled with ICS (≥200 μg/d FP or equivalent) and history of severe asthma attacks in the last 2 years	Rates of asthma attacks requiring either oral steroids or doubling inhaled corticosteroids, were significantly reduced at both 24 weeks (remained on their existing maintenance steroids) and 52 weeks (steroid adjustment made) were 31% lower (treatment) versus 43% lower (control) Analysis of the severe asthma cohort (inadequately controlled with high‐dose ICS (≥500 μg/d FP or equivalent) + LABA ± additional controller‐ Omalizumab, *n* = 159; placebo, *n* = 76), showed that the rate of asthma attacks was reduced by 34% versus placebo over 24 weeks and by 50% over 52 weeks
Busse et al.^24^ ICATA study Double‐blind randomized, placebo‐controlled, parallel‐group muti‐centered trial	Four hundred nineteen participants Omalizumab *n* = 208 Placebo *n* = 211 Age 6–20 years	Inner city children with persistent/uncontrolled allergic asthma (hospital visit in last year), positive skin test for a perennial allergen, and total serum levels of IgE between 30 and 1300 IU/ml.	Treatment group showed 24.5% reduction in number of days with asthma related symptoms—from 1.96 to 1.48 days per 2 weeks, compared to placebo. Reduced numbers showing at least one asthma attack during trial period—30.3% (treatment) versus 48.8% (control) Asthma related hospitalizations were lower—1.5% (treatment) versus 6.3% (control). Inhaled corticosteroid requirements were lower‐ 663 μg/day (treatment) versus 771 μg/day (placebo) budesonide equivalent dose. Significant improvements were observed in patients sensitized to cockroaches, with a reduction in number of symptomatic days, dose of glucocorticoids needed, and rate of asthma attacks *Post hoc* analysis showed a dramatic decrease in seasonal surge (spring and autumn) in asthma symptoms and attacks in the treatment group, indicating a possible link between IgE and upper respiratory tract infection‐related attacks.
Teach et al.^25^ 3‐arm, randomized, double‐blind, double placebo‐controlled, multicentre clinical trial	Five hundred thirteen children 453 participants omalizumab *n* = 223, ICS boost arm *n* = 155, placebo *n* = 75) Age 6–17 years	Inner city children with uncontrolled asthma with ICS (≥200 μg/d FP or equivalent), one or more asthma attacks (requiring systemic corticosteroids) or hospitalization within the prior 19 months, positive skin test to perennial allergen and total serum IgE level 30–1300 IU/ml.	A reduction in asthma attacks was observed during the autumn surge period—11.3% (omalizumab) versus 21% (placebo). No difference was seen in the reduction of autumn surge asthma attacks between omalizumab (8.4%) and ICS boost (11.1%). Larger IFN‐α responses to rhinovirus in the treatment group and within the omalizumab group were associated with fewer attacks (OR 0.14)

For children with severe.^26^, ^27^ and moderate to severe.^28^ asthma, meta‐analyses and a systematic review.^29^ found that omalizumab use was associated with reduced numbers of asthma attacks per year, improved quality of life, reduced use of salbutamol as a rescue medication and reduced doses of ICS compared to placebo. Despite the heterogeneity of the studies included in these meta‐analyses, there is still sufficient evidence to conclude that omalizumab can produce sustained improvements in symptom and quality of life scores.^30^

Chipps et al.^31^ reviewed clinical trials and 7 years of real world data, demonstrating that omalizumab treatment was associated with improved asthma symptom control, sustained reduction in ICS use, reduced rates of asthma attacks and unscheduled hospital visits, in cases of moderate and severe pediatric asthma. There is also strong evidence that omalizumab has anti‐inflammatory properties, demonstrated by sustained maintenance of FeNO reduction, even with significant reduction of ICS dose.^32^

Deschildre et al. reported follow‐up data after the use of omalizumab in children aged 6–18 years. At 1 year, asthma attacks were reduced by 72% and hospitalization by 88.5%. 67% of children achieved adequate symptom control after starting treatment and reduced ICS use by at least 30%, and average forced expiratory volume (FEV_1_) increased by 4.1% from baseline.^33^ After 2 years, the rate of severe asthma attacks dropped further (83% below first year) and no study participants required hospitalization for asthma. However, there was no additional improvement in symptom control, reduction in ICS use or spirometry. 20.7% of participants stopped omalizumab during the second year, mainly due to delayed side effects or lack of additional symptom improvement.^34^

Although omalizumab produces a sustained reduction in total free IgE throughout treatment, especially in patients with uncontrolled asthma,^31^ this biomarker is not a meaningful predictor of treatment response. However, there is evidence that children aged 12 and older with higher levels of Th‐2 markers (FeNO, periostin and blood eosinophil counts) had better outcomes with treatment,^35^ though no comparable evidence yet exists for younger children. Serum periostin, which is thought to be a reliable biomarker in adults, is elevated in children, as it is also produced by growing bones and is therefore not useful.

### 4.3 Safety profile

Omalizumab has been used since 2003 in the USA and there is extensive safety data available.^22^, ^24^ Rare reactions include a delayed allergic type III reaction (typically observed between days 1–5 after treatment) which may be caused by immune complex formation and deposition due to development of antibodies against omalizumab. Symptoms include arthritis/arthralgias, rash, fever and lymphadenopathy, which can be treated with antihistamines and corticosteroids.^36^ Another rare reaction is systemic hypereosinophilic syndrome or Churg‐Strauss syndrome.^37^ Symptoms include marked eosinophilia in the presence of vasculitic rash, worsening pulmonary symptoms, cardiac complications, and/or neuropathy.^37^ In clinical trials, some participants also experienced reduction in platelet counts, but this was not persistent and was not associated with bleeding episodes or a decrease in hemoglobin.^38^ In addition, IgE is an essential component of the immune response protecting against helminth infections and therefore medications lowering IgE levels should be used with caution in helminth endemic areas (a consideration for all anti Th‐2 medications).

## 5 MEPOLIZUMAB

### 5.1 Mechanism of action

Currently, three monoclonal antibodies have been developed to target the IL‐5 signaling pathway. Mepolizumab and reslizumab bind interleukin 5 (IL‐5) directly, while benralizumab targets the IL‐5 receptor alpha (IL‐5Rα) subunit, both of which result in apoptosis of eosinophils and basophils (Figure 2). However, currently only mepolizumab (Nucala, GlaxoSmithKline) has been licensed in the UK as an add‐on treatment for severe refractory eosinophilic asthma in children aged 6 years and older.^39^

### 5.2 Indications

Mepolizumab is a humanized IgG1 monoclonal antibody which binds to IL‐5, and blocks IL‐5 binding with interleukin‐5 receptor alpha (IL‐5Rα), thereby inhibiting eosinophilic inflammation (Figure 2).^40^ The dose is age dependent; 40 mg for children aged 6–11 years, and 100 mg in children ≥12 years, administered 4‐weekly subcutaneously. It is used as an add‐on therapy in children aged 6 years and over with severe refractory eosinophilic asthma, which is defined as either^1^ a blood eosinophil count of ≥300 cells/µl in the last 12 months, and either (i) at least 4 attacks requiring treatment with systemic corticosteroids in the previous 12 months, or (ii) oral corticosteroids daily of at least the equivalent of prednisolone 5 mg/day over the previous 6 months; or^2^ a blood eosinophil count of ≥400 cells/µl in the last 12 months and at least three attacks requiring systemic steroids during the previous 12 months (Table 5). Mepolizumab has been shown to reduce both blood and sputum eosinophil counts, and to reduce oral glucocorticoid requirement.^41^, ^42^ NICE recommends a review after 12 months of treatment, before deciding whether to continue, either a 50% reduction in attacks or reduction in maintenance oral corticosteroids is required for continuation. Table 2 summarizes the main studies looking at the efficacy of mepolizumab.

**Table 2:** Summary of main studies looking at the efficacy of mepolizumab.

Study	Number of participants and age	Methods/Inclusion criteria	Summary of Evidence
Ortega et al.^43^ MENSA study Multicenter, randomized, double‐blind, double‐placebo, phase 3, placebo‐controlled trial	576 participants 75 mg mepolizumab intravenously *n* = 191 100 mg mepolizumab subcutaneously *n* = 194 Placebo *n* = 191 Age 12–82 years	Under age of 18 years: FEV1 < 90% predicted or FEV1/FVC < 0.8. BDR > 12%, with FEV1 variability ≥20% in the past 12 months, in addition to >2 asthma attacks in the previous year that were treated with systemic glucocorticoids while on ≥880 μg/d FP or equivalent + additional controller, as well as an eosinophil count >150 cells/µl in peripheral blood at screening time or >300 cells/µl in last 12 months.	Both methods of administration were equally effective Reduction in rate of asthma attacks: 47% (intravenous) and 53% (subcutaneous) versus placebo Reduction in unscheduled hospital visits: 32% (intravenous) and 61% (subcutaneous) versus placebo Mean FEV1 increase from baseline pre‐bronchodilator: 100 ml (intravenous) and 98 ml (subcutaneous) versus placebo Mean FEV1 increase from baseline pre‐bronchodilator: 146 ml (intravenous) and 138 ml (subcutaneous) versus placebo Improvement in quality‐of‐life score (numerical decrease): 6.4 (intravenous) and 7.0 (subcutaneous) versus placebo (change of four points was considered clinically significant) Improvement in asthma control score (numerical decrease): 0.42 (intravenous) and 0.44 (subcutaneous) versus placebo (0.5 point change was considered clinically significant) Reduction in eosinophil counts (nadir by 12 weeks) by 83% (intravenous) and 86% (subcutaneous) versus placebo and maintained throughout the 12‐month period of study
Bel et al.^41^ SIRIUS study Multicenter, randomized, placebo‐controlled, double‐blind, parallel‐group study with an optimization of oral steroid phase	139 participants Mepolizumab *n* = 69 Placebo *n* = 66 Age 16–74 years	≥6 months systemic corticosteroids as preventer (5–35 mg/day prednisolone or equivalent) and blood eosinophil count >150 cells/µl during steroid optimization phase or >300 cells/µl in last 12 months. Preventer therapy included high dose ICS and an additional therapy.	Treatment group: More patients achieved 90%–100% reduction in oral corticosteroid (23% vs. 11% in placebo) and 70%–90% reduction in oral corticosteroid (17% vs. 8% in placebo). Overall, mepolizumab treatment resulted in 2.39 times greater likelihood of a reduction in glucocorticoid doses Median reduction of 50% in glucocorticoid doses from baseline in treatment group, compared to 0% in placebo Despite oral steroid reduction, the numbers of asthma attacks per year were 32% lower than controls (1.44 vs. 2.12) Improvement in asthma control score ACQ‐5 (numerical decrease) was 0.52 versus placebo (0.5 point change was considered clinically significant)
Jackson et al.^44^ MUPPITS‐2 Randomized, double‐blind, placebo‐controlled, parallel‐group trial	290 participants Mepolizumab *n* = 146 Placebo *n* = 144 Age 6–17 years	Children from minority ethnic groups in socially deprived areas in the USA with ≥2 attacks in the last year and blood eosinophil count ≥150 cells/µl	Mean number of asthma attacks per year in the mepolizumab group was 0.96, versus 1.30 (placebo)

Although mepolizumab was licenced for use in children aged 6 years and above, the main body of evidence for its use has been criticized on the basis that it only included 34 12–17 year old children across four studies (Table 2). REALITI‐A, the largest prospective, real‐world analysis of mepolizumab efficacy, showed significant reductions in oral corticosteroid maintenance dose and asthma attacks, with no significant safety concerns identified. However, this study only included adults, nearly 40% of whom were on maintenance oral corticosteroids (indicating a high baseline level of severity).^45^

Two meta‐analyses of real world effectiveness of mepolizumab in both adults and adolescents showed reductions in attacks and hospitalizations, as well as clinically significant symptom control improvements, despite reductions in oral glucocorticoid use, as well as reductions in peripheral blood eosinophil count and FeNO.^46^, ^47^ Although baseline blood eosinophil count appears to be a reliable biomarker to predict response to mepolizumab, there are several other factors to consider, especially in children, as we discuss later.^41^, ^43^, ^46^, ^48^ An extension of the MENSA study showed that sputum and blood eosinophil counts returned to pretreatment levels within 3 months of treatment cessation, and that attack frequency worsened within 3–6 months.^49^

Gupta et al. undertook a study in a small cohort of 6–11 year olds to evaluate bioavailability and dosage adjustment of mepolizumab in children, during which no safety concerns were observed.^50^, ^51^ Blood eosinophil count reduced after treatment commenced, correlating with symptom improvement, but lung function did not significantly improve.^51^ These benefits were sustained over a 1‐year observation period, during which asthma attacks were reduced by over 50% in 80% of children.^50^ However, this was an open label, non‐randomized study which was not powered for efficacy and a Type 1 error cannot be excluded.

MUPPITS‐2 is an important randomized double‐blind placebo controlled trial carried out in children (Table 2), which overall reported a small (but statistically significant) difference in reductions of asthma attacks for children treated with mepolizumab (27% reduction) compared to placebo, a much less favorable outcome than in adults (50% reduction).^44^ This study is a powerful reminder that there are differences between the pathophysiology of pediatric and adult asthma and that results in children may not have the same clinical benefit as in adults. Nasal transcriptomics analyses showed that although children with a type‐2 high, eosinophilic predominant signature benefitted from mepolizumab, there was a sub‐group with an epithelial/remodeling predominant profile whose symptoms worsened on mepolizumab. The MUPPITS‐2 trial also had limitations. The children were specifically recruited from ethnic groups (Black and Hispanic) known to have higher exposure to asthma‐exacerbating factors such as air pollution and secondary tobacco smoke, and therefore may not have maximally benefited from mepolizumab treatment.^52^ Another reason for this discrepancy could be that IL‐5 levels in bronchoalveolar lavage increase with age, and therefore an anti‐IL‐5 agent may be less effective in younger children and more efficacious in older age groups with higher IL‐5 levels. This study is notable for using objective markers of efficacy using airway transcriptomic profiling to identify and analyze treatment responses.

### 5.3 Safety profile

A systematic review of 13 studies for the currently available anti‐IL‐5 therapies (four studies included children 12–17 years old, with small numbers recruited and no separate analysis), showing that mepolizumab had a better safety profile than reslizumab and benralizumab.^40^ There was no clear evidence of increased infection or neoplasm in children aged 6–11 years, with the limitation that this study was conducted in a small number of children (*n* = 36), and there was no control group for comparison.^53^

### 5.4 Benralizumab

Benralizumab (Fasenra, Astra Zeneca) is a biologic that blocks the IL‐5 receptor, and is not licenced in the UK, but is used in children in the USA. There was a higher rate of discontinuation of benralizumab, compared to placebo, due to adverse events^40^ and the TATE study, which did not assess efficacy in children, showed that in 6–14 year olds on benralizumab, 78.6% of children experienced side effects.^54^ Therefore, benralizumab is less well tolerated than mepolizumab, the alternative modulator of the IL‐5 pathway available in children.

## 6 DUPILUMAB

### 6.1 Mechanism of action

IL‐4 and IL‐13 are two structurally similar cytokines which play key roles at multiple stages of the allergic response, including by enabling differentiation of naïve T‐helper cells, upregulating MHC class II and Fcϵ receptor expression, promoting secretion of IgE, inducing B cell class switching, activating mast cells, and supporting proliferation of eosinophils and basophils. IL‐4 and IL‐13 are therefore promising targets for therapeutic intervention in allergic diseases.^55^

Dupilumab (Dupixent, Sanofi Biotechnology) is a human monoclonal antibody which blocks IL‐4Rα, the shared receptor for both IL‐4 and IL‐13 (Figure 2).^56^ Because IL‐4 and IL‐13 also increase production of nitric oxide by airway epithelial cells during eosinophilic inflammation, FeNO is a very useful biomarker for identifying children who may benefit from dupilumab.^57^

### 6.2 Indications

Dupilumab was initially licensed for atopic eczema and is now licenced for use in the UK in children age 6 years and over, with severe type‐2 mediated asthma as an add‐on therapy. The dosing is calculated by age and body weight in the 6–11 year‐old group. In children over 12 years, it is prescribed as a single initial dose of 400 mg, administered subcutaneously as two consecutive 200 mg injections at different sites, followed by 200 mg every 2 weeks.^58^ Criteria for dupilumab are blood eosinophil count ≥150 cells/µl, FeNO ≥25 parts per billion and poorly controlled asthma (≥4 asthma attacks), in the last 12 months (Table 5). NICE stipulates that dupilumab can only be prescribed if the child is either not eligible for or exhibited an undefined poor response to mepolizumab, the latter defined as less than 50% reduction in severe asthma attacks over 12 months. However, given the better results of VOYAGE compared to MUPPITS‐2, this may not be fully justified. NICE recommends a review after 12 months of initiating treatment, which can be continued if the rate of severe attacks (need for short course of oral corticosteroids) has been halved. Table 3 summarizes the main pediatric studies looking at the efficacy of dupilumab.

**Table 3:** Summary of main pediatric studies looking at the efficacy of dupilumab.

Study	Number of participants and age	Methods/Inclusion criteria	Summary of Evidence
Bacharier et al.^56^ VOYAGE study Phase 3, randomized, double‐blind, placebo‐controlled trial	408 participants Dupilumab *n* = 273 Placebo *n* = 135 Age 6–11 years	Group 1 (type 2 inflammatory phenophyte): Blood eosinophil count ≥150/µl or FeNO ≥20 ppb (baseline of eosinophil <150/µl kept at 20% of population) Group 2: Blood eosinophils ≥300 cells/µl. Subcutaneous dupilumab weight‐based dosing (100 mg if ≤30 kg, 200 mg >30 kg)	Dupilumab reduced the number of asthma attacks per year by 59.3% (relative risk reduction) Annual rates of asthma attacks: Group 1 on dupilumab: 0.31 versus placebo 0.75. Group 2 on dupilumab: 0.24 versus placebo 0.67 Larger reductions were observed in Group 2 (64.7%), and Group 1 participants with eosinophil counts ≥150 cells/µl) (61%) FeNO ≥20 ppb‐ relative reduction of 54.2% versus placebo in Group 1 Percentage of participants suffering no attacks during the 52 week trial: Group 1: 77.1% (dupilumab) versus 59.6% (placebo), Group 2: 79% (dupilumab) versus 58.3% (placebo). Reduction in systemic corticosteroid requirement in dupilumab versus placebo was 59.3% in Group 1 and 66% in Group 2 Improvements in FEV1 (indicating an improvement in lung function) in Group 1 was 10.5% (dupilumab) versus 5.3% (placebo) and similar in Group 2 Reduction in asthma control scores (ACQ‐7‐IA) for Group 1 was −1.33 (dupilumab) versus −1.00 (placebo) and for Group 2 was −1.34 (dupilumab) versus −0.88 (placebo) During the treatment period, there was a larger decrease in type 2 inflammatory markers (mean FeNO and total serum IgE) compared to placebo, which supports their use as biomarkers to monitor response to treatment
Castro et al.^59^ QUEST trial randomized, double‐blind, placebo‐controlled, parallel‐group trial	1902 participants Group 1: Dupilumab 200 mg *n* = 631 Group 2: Placebo 1.14 ml *n* = 317 Group 3: Dupilumab 300 mg *n* = 633 Group 4: Placebo 2.00 ml *n* = 321 Age ≥12 years	On medium‐to‐high‐dose inhaled glucocorticoid (≥500 μg per day of fluticasone propionate or equivalent) and up to two other preventers. FEV1 at baseline ≤80% predicted (or ≤90% predicted in 12–17 year olds); ≥12% FEV1 reversibility; ACQ‐5 score ≥1.5; unscheduled hospital visit or use of ≥3 days of systemic steroids in the last year.	Number of asthma attacks per year: Group 1: 0.46 versus Group 2: 0.87 (matched placebo) and Group 3: 0.52 versus Group 4: 0.97 (matched placebo) In participants with eosinophil counts of ≥300 cells/µL, number of attacks per year were significantly improved (0.37 in Group 1 and 0.40 in Group 3) compared to matched placebos (1.08 in Group 2 and 1.24 in Group 4), improvement of FEV1 at 12 weeks was 0.43 L in Group 1 and 0.47 L in Group 3 versus matched placebos (0.21 L in Group 2 and 0.22 L in Group 4, respectively). In participants with eosinophil counts of 150‐300 cells/µl, number of attacks per year were also significantly improved (0.56 in Group 1 and 0.47 in Group 3 300 mg) compared to matched placebos (0.87 in Group 2 and 0.84 in Group 4), improvement of FEV1 at 12 weeks was 0.28 L in Group 1 and 0.25 L in Group 3, versus matched placebos (0.17 L in Group 2 and 0.25 L in Group 4, respectively). In those with FeNO ≥50 ppb, FEV1 improvement of 0.30 L was noted in Group 1 compared to matched placebo (Group 2) and 0.39 L in Group 3 compared to Group 4 matched placebo. Dupilumab improved ACQ‐5 score from as early as 2 weeks after starting treatment, asthma quality of life questionnaire score (vs. placebo), as well as morning and evening peak expiratory flow and symptom scores. 46.8% fewer unscheduled hospital visits in dupilumab group compared to placebo.
Rabe et al.^60^ VENTURE trial phase 3, randomized, double‐blind, placebo‐controlled trial	210 participants Dupilumab *n* = 103 Placebo *n* = 107 Age ≥12 years	On regular oral corticosteroids (5–35 mg of prednisolone or equivalent) in the last 6 months, on high‐dose inhaled ICS (>500 μg per day of fluticasone propionate or equivalent), and up to two other preventers. FEV1 at baseline ≤80% predicted (or ≤90% predicted in 12–17 year olds); ≥12% FEV1 reversibility.	48% of patients were able to completely cease using prednisolone, compared to 25% in the placebo group, as well as a 70.1% reduction in glucocorticoid administration. Although the placebo group also showed a reduction of 41.9% in glucocorticoid use, this was attributable to the effects of improved compliance to treatment in a clinical trial setting, and despite this context, dupilumab still enabled significantly greater reductions in the steroid burden. Overall, 80% of trial participants managed to at least halve their glucocorticoid dose requirement (compared to 50% in the placebo group). Despite the significant reductions in steroid use with dupilumab, the rate of severe asthma attacks was simultaneously reduced by 59% in the treatment group, which was accompanied by an increase in FEV1 by 0.22 L overall, with better asthma control scores. Greater benefit in participants with higher blood eosinophil counts, where sub‐analysis of patients with an eosinophil count of ≥300 cells/µl produced a 71% reduction in the rate of severe attacks, and an increase in FEV1 by 0.32 L, compared to matched placebo groups.

*Post‐hoc* analysis of the QUEST study, analyzing efficacy in adolescents aged 12–17 years old (109 (5.7%) of total participants), showed similar results to the adult studies, with improvements in spirometry (FEV_1_) and annual attack rates for patients with elevated Th‐2 inflammation biomarkers, as well as improvements in asthma control and quality‐of‐life scores.^61^

The EXCURSION study, which is an open‐label extension of the VOYAGER study, looked at the safety and efficacy of dupilumab in children between 6 and 11 years, over 2 years, showing similar results. 91% of children on dupilumab had no asthma attacks during the 1‐year follow‐up phase of this study, and of those who had attacks, 83% had only one attack. The dupilumab treatment group also showed reductions in type 2 inflammatory markers such as IgE and need for systemic corticosteroids. The treatment group also demonstrated improvements in spirometry results (pre bronchodilator FEV_1_) in the first 2 weeks of starting treatment and this is sustained throughout the treatment period.^62^ It should be noted that the annualized attack frequency in VOYAGE in the placebo group was 2.16/year which fell with placebo to 0.67/year (the fall with dupilumab was significantly greater‐ 0.24/year)! This underscores (a) the need for placebo‐controlled trials, and (b) how much can be achieved by improving basic care (in this case, trial effect).

### 6.3 Safety profile

Dupilumab has been shown to be associated with a rise in blood eosinophil count in several studies. However, longer term monitoring showed that the hypereosinophilia was transient and self‐limiting and did not necessitate treatment discontinuation.^56^, ^62^ The rate of parasitic infection was reported at around 2% of children on dupilumab, and was not considered to be serious or severe, and an even smaller number were associated with eosinophilia.^62^ Dupilumab treatment was also associated with higher incidence of upper respiratory tract viral infections (12.2% in treatment group vs. 9.7% in placebo).^56^ Similarly, the EXCURSION study showed that nasopharyngitis, pharyngitis, and upper respiratory tract infection were more common in the treatment group compared to placebo.^62^

Ocular side effects, such as increased risk of dry eyes and conjunctivitis, have also been reported with dupilumab use and therefore should also be discussed with parents and patients before commencing treatment. Overall, dupilumab has been shown to be well tolerated and to have a very good safety profile in the pediatric population.^62^

## 7 TEZEPELUMAB

Tezepelumab is the newest approved biologic for use in children with severe asthma aged ≥12 years in the UK. It is a monoclonal antibody that blocks the activity of thymic stromal lymphopoietin (TSLP), an IL‐7 like epithelial cell‐derived cytokine (alarmin), that plays a key role in the immune response to proinflammatory inhaled stimuli such as allergens and infections (Figure 2).^63^, ^64^ TSLP is involved in the activation of multiple inflammatory signaling pathways, including JAK/STAT and the PI‐3 kinase pathway, and plays a major role in stimulating downstream inflammatory cascades through its effects on dendritic cells, T and B cells, and innate lymphoid cells, as well as stimulating the release of antigen specific Th‐2 cytokines. This central role of TSLP makes it potentially a useful target to treat allergic diseases, including asthma.^64^ In addition, TSLP is also thought to play an important role in the early stages of the non‐Th‐2 pathway, making it a potential target for addressing the unmet need of patients with non‐eosinophilic, nonallergic asthma.^65^ Higher TSLP airway expression has been correlated with severity of asthma.^66^

### 7.1 Indications

Tezepelumab (Tezpire, Astra Zeneca) is licenced in the UK for children aged 12 years and older with severe uncontrolled asthma, despite being treated with high dose ICS and one other preventer (LABA, LTRA, theophylline). There is no biomarker threshold for tezepelumab, making it suitable for children with STRA, who are not eligible for the other three biologics. NICE guidelines require the child to have had ≥3 attacks in the previous year or requiring oral corticosteroids for maintenance, to qualify for Tezepelumab (Table 5). Tezepelumab is dosed at 210 mg every 4 weeks subcutaneously. NICE recommends that treatment with tezepelumab should be reviewed at 12 months and stopped if the number of severe asthma attacks or the requirement of oral corticosteroids are not reduced by half.^67^ Table 4 summarizes the main studies looking at the efficacy of tezepelumab.

**Table 4:** Summary of main studies looking at the efficacy of tezepelumab.

Study	Number of participants and age	Methods/Inclusion criteria	Summary of Evidence
Corren et al.^68^ PATHWAY study multicentre, placebo‐controlled, parallel‐group, double‐phase 2 trial	550 participants Group 1: Low dose tezepelumab *n* = 138, Group 2: Medium dose tezepelumab *n* = 137 Group 3: High dose tezepelumab *n* = 137 Group 4: Placebo *n* = 138 Age 18–75 years	Current nonsmokers, poor symptom control on LABAs and a medium‐high dose (≥250 μg/day fluticasone or equivalent), ≥2 asthma attacks that required systemic steroids, or one hospitalization for asthma during the last 12 months. Baseline forced expiratory volume in 1 s (FEV1) 40%–80% predicted and BDR or ≥12% and ≥200 ml, and ACQ‐6 score ≥1.5 during screening.	Annual rate of asthma attacks Group 1: 0.27, Group 2: 0.20 and Group 3: 0.23 compared to placebo 0.72 (reduction of 62% in Group 1, 71% in Group 2 and 66% in Group 3 compared to placebo) In moderate asthma category‐ annual rate of asthma attacks Group 1:0.20, Group 2: 0.15, Group 3: 0.20 compared to placebo 0.38 (reduction of 48% in Group 1, 60% in Group 2 and 48% in Group 3 compared to placebo) In severe asthma category‐ annual rate of asthma attacks Group 1:0.35, Group 2: 0.26, Group 3: 0.27 compared to placebo 1.12 (reduction of 70% in Group 1, 77% in Group 2 and 76% in Group 3 compared to placebo) Improvement in baseline FEV1 at 52 weeks‐ Group 1: 0.12 L, Group 2: 0.13 L and Group 3: 0.15 L compared to placebo Biomarkers: reduction in blood eosinophil count, FeNO and total IgE with tezepelumab
Menzies‐Gow et al.^69^ NAVIGATOR study multicenter, randomized, double‐blind, placebo‐controlled phase 3 trial	1061 participants Tezepelumab *n* = 529 Placebo *n* = 532 Age 12–80 years old	Medium to high dose ICS (≥500 μg fluticasone or equivalent) and one additional preventer. Baseline forced expiratory volume in 1 s (FEV1) < 80% predicted (<90% predicted for ages 12–17 years). BDR ≥ 12% and ≥200 ml. Minimum of 2 asthma attacks in 12 months requiring ≥3 days of oral corticosteroid or hospitalization.	Number of asthma attacks per year: 0.93 in tezepelumab and 2.10 in placebo group Sub analysis in participants based on blood eosinophil count: (i) ≤300 cells/µl: number of asthma attacks per year 1.02 in tezepelumab compared to 1.73 in placebo group (ii) ≤150 cells/µl: number of asthma attacks per year 1.04 in tezepelumab compared to 1.70 in placebo group 43.8% in tezepelumab group and 60.1% in placebo group had at least one attack during the 52 weeks trial period. Baseline FEV1 increased by 0.23 L in tezepelumab group compared to 0.09 L in the placebo group Significant improvements in asthma symptom scoring questionnaires (ACQ‐6, AQLQ(S) + 12, ASD), reduction in baseline eosinophil count and FeNo levels were noticed in the tezepelumab group after 2 weeks and were sustained throughout treatment, compared to placebo Rate of unscheduled hospital visits was 0.06 in tezepelumab group compared to 0.28 in placebo.
Corren et al.^70^ Pooled analysis of PATHWAY and NAVIGATOR study	1334 participants Tezepelumab *n* = 665 Placebo *n* = 669 Age PATHWAY 18–75 years old NAVIGATOR, 12–80 years old	As above for each individual study	Annual rates of asthma attack reduced by 60% with tezepelumab Biomarker specific outcomes for annual rates of asthma attack reduction (compared to placebo): FeNO <25 ppb subgroup‐ 40% reduction Blood eosinophil counts ≥450 cells/μl subgroup −78% reduction Evidence of aeroallergen senitization subgroup‐ 62% reduction Without evidence of aeroallergen senitization subgroup‐ 54% reduction Combination of blood eosinophil counts of ≥300 cells/μl and evidence of aeroallergen senitization subgroup‐ 71% reduction. “Triple Th‐2 low” subgroup (blood eosinophil counts <150 cells/μl, FeNO levels <25 ppb, and without aeroallergen senitization‐ 34% reduction Reduction in unscheduled hospital visits/admissions by 79% overall in treatment group (reduction ranged between 60%–94% in all the combinations of biomarkers subgroups above) Prebronchodilator FEV1 at 1 year improved by 0.22 L in treatment group versus 0.09 L in placebo group
Menzies‐Gow et al.^71^ DESTINATION study. Phase‐3, randomized, double‐blind, placebo‐controlled, long‐term extension study of participants in NAVIGATOR and SOURCE trials	Total 951 participants (*n* = 827 from NAVIGATOR study and *n* = 124 from SOURCE study) Tezepelumab *n* = 602 Placebo *n* = 607 Participants on placebo in parent study were re‐randomized 1:1 for this study Age 12–80 years old	As above for each study	Well tolerated with a very good safety profile for up to 2 years Lower incidence of adverse events and serious adverse events, in tezepelumab group compared to placebo. Condition specific incidence of serious adverse events ‐ chest related conditions lower in treatment group versus placebo ‐ cardiac related events higher in treatment group versus placebo (note: all had at >2 cardiac risk factors and 44% had a cardiac diagnosis) Clinically significant reductions in annual asthma attacks and improvement in spirometry, and quality of life, were maintained during the observation period of up to 2 years.

The SOURCE study.^72^ recruited 150 participants between the ages of 18 and 80 years to investigate the reduction of oral steroids that could be achieved during tezepelumab treatment without losing asthma control. A significant reduction in steroid requirement was achieved in participants with blood eosinophil counts of ≥150 cells/µl, but not in those with ≤150 cells/µl.

A meta‐analysis of six RCTs included analysis of 2667 participants showed that tezepelumab was associated with an improvement in FEV_1_, a reduction in the number of asthma attacks per year and a reduction in FeNO compared to placebo, with a good safety profile. However due to the heterogeneity of the trials included, and to the multiple targets of the mechanism of action of tezepelumab, this meta‐analysis could not identify particular subgroups (Th‐2 high or low) who would benefit more from this therapy.^73^ This was addressed by the post hoc analysis of the PATHWAY and NAVIGATOR studies, which showed that tezepelumab was more efficacious compared to placebo regardless of baseline biomarker levels, including in the “Triple Th‐2 low” subgroup (blood eosinophil counts <150 cells/μl, FeNO levels <25 ppb, and without aeroallergen senitization).^70^ This supports the use of tezepelumab in cases of severe asthma, without biomarker limitations. However, caution still needs to be exercised in extrapolating this data to children, as the mean age of participants in those studies was 50 years old, and it is known that the Th‐2 low phenotype is more prevalent in the adult population and may be mechanistically different to Th‐2 low in children.

### 7.2 Safety profile

Although currently only limited safety data exists for use in children, the DESTINATION study^69^, ^71^ is an ongoing long‐term extension study from the NAVIGATOR and SOURCE cohort, in participants between 12 and 80 years old. This study demonstrated a very good safety profile, with the common side effects being nasopharyngitis and upper respiratory tract infections.^71^ No serious adverse events reported for tezepelumab over 2 years and similar safety data was observed in the pooled analysis of the PATHWAY and NAVIGATOR clinical trials.^68^, ^69^, ^70^, ^74^

## 8 SUMMARY OF NOVEL BIOLOGICS, ELIGIBILITY AND SIDE EFFECTS

All five biologics approved for use in children (Table 5) have very good safety profiles. The common and drug specific side effects for each biologic is listed in Table 6.^75^ Due to a 1:1000 risk of anaphylaxis reaction, treatment is initially commenced in hospital, before later being continued as home administration.

**Table 5:** Summary of licenced biologics in children and eligibility criteria.

Biologic	Age licenced for use in the UK	Eligibility criteria	Dosing
**Omalizumab** (anti‐IgE humanized monoclonal antibody)	≥6 years	(1) Confirmed severe, persistent, IgE mediated allergic asthma with: serum IgE within 30–1500 IU/ml evidence of senitization to at least one perennial aeroallergen, regardless of eosinophil count (2) ≥4 courses of oral corticosteroids in the last 12 months	Subcutaneous injection every 2–4 weeks. Dose determined by baseline serum IgE and body weight
**Mepolizumab** (anti‐IL‐5 humanized monoclonal antibody)	≥6 years	Confirmed severe refractory eosinophilic asthma with either: (1) a blood eosinophil count of ≥300 cells/µl in the last 12 months, and either (i) ≥4 attacks requiring treatment with systemic corticosteroids in the previous 12 months, or (ii) oral corticosteroids daily of at least the equivalent of prednisolone 5 mg/day over the previous 6 months; or(2) a blood eosinophil count of ≥400 cells/µl in the last 12 months and at least 3 attacks requiring systemic steroids during the previous 12 months.	Subcutaneous injection every 4 weeks. Dose is age dependent; 6–11 years: 40 mg ≥12 years: 100 mg
**Dupilumab** (anti‐IL‐4Rα humanized monoclonal antibody)	≥6 years	Confirmed severe Th‐2 mediated asthma with either(1) Blood eosinophil count ≥150 cells/µl **OR** FeNO ≥25 parts per billion **and** (2) Poorly controlled asthma (≥4 asthma attacks), in the last 12 months.	Subcutaneous injection. 6–11 years‐dose calculated by body weight ≥12 years‐ single initial dose of 400 mg, followed by 200 mg every 2 weeks
**Tezepelumab** (anti‐TSLP humanized monoclonal antibody)	≥12 years	Severe uncontrolled asthma‐ no biomarker threshold ≥3 attacks in the previous year or requiring oral corticosteroids for maintenance	Subcutaneous injection 210 mg every 4 weeks.
**Benralizumab** (IL‐5 receptor blocker humanized monoclonal antibody) Only licenced for use in the USA and not in the UK	≥6 years (USA)	Severe eosinophilic asthma (on regular medium/high dose ICS and LABA, ± oral corticosteroids or another preventer) with ≥2 attacks in the previous year, requiring oral or systemic corticosteroid treatment, and reduced lung function (FEV1) at baseline	Subcutaneous injection, every 4 weeks for the first 3 doses and every 8 weeks thereafter. Dose is weight dependent. Weight ≥35 kg 30 mg, Weight <35 kg 10 mg.

**Table 6:** Summary of side effects of biologics licensed in children (UK and USA).

Biologic	Side effects
Omalizumab	Fever, abdominal pain, headache, skin reactions (irritation/itching/erythema), arthralgia, dizziness, fatigue, ear pain, increased risk of upper respiratory tract infections and nasopharyngitis, nose bleeds and anaphylaxis. Drug specific side effect: Delayed allergic type III reaction (serum sickness reaction), thrombocytopenia, and eosinophilic granulomatosis with polyangiitis (Churg‐Strauss syndrome), which is usually associated with the reduction of oral corticosteroids.
Mepolizumab	Abdominal pain, back pain, fever, eczema, headache, hypersensitivity reactions, increased risk of infection, nasal congestion.
	Drug specific side effect: Helminthic infections
Dupilumab	Arthralgia, hypersensitivity reactions, ocular side effects (dry eyes, conjunctivitis, blepharitis & pruritis), increased risk of upper respiratory tract viral infections and nasopharyngitis
	Drug specific side effects: Helminthic infections, transient hypereosinophilia, and eosinophilic granulomatosis with polyangiitis (Churg‐Strauss syndrome), which is usually associated with the reduction of oral corticosteroids.
Tezepelumab	Arthralgia, skin reaction, headache, increased risk of upper respiratory tract viral infections and nasopharyngitis, hypersensitivity reactions and back pain
Benralizumab	Hypersensitivity reactions, increased risk of upper respiratory tract viral infections and nasopharyngitis, and headache
	Drug specific side effects: Helminthic infections

## 9 OVERALL CONSIDERATIONS FOR TH‐2 BIOLOGIC THERAPY

The common assumption is that eosinophils are pathogenic in asthma and lead to exacerbation of chronic *airway* inflammation, triggering asthma attacks, and should be vigorously obliterated. *Blood* eosinophil counts are used to determine eligibility for biologics in severe asthma, including discrete cut‐offs in both adults and children. However, blood eosinophil levels are much higher in “normal” children than adults, and gender differences have also been observed.^76^ Eosinophil counts are elevated in atopic diseases (not specific to asthma), parasitic infections and by environmental factors, including living conditions and smoking status.^76^ It seems illogical to use adult cutoffs of blood eosinophil counts as a biomarker to determine eligibility for biologics in children. We do not know if blood eosinophil counts are stable over time in children, or how many measurements are needed to define Th‐2 low asthma, and there is also little evidence for any relationship between blood and airway eosinophils in children.^77^ These caveats may explain the surprisingly small improvements in asthma attacks in the MUPPITS‐2 trial compared to adult studies, as the blood eosinophil count of ≥150 cells/µL used to select the eligible population for mepolizumab may have been too low to select for children who truly have airway eosinophilia, because this level is actually *below* the mean level in normal younger children.

The second question relates to whether eosinophils are always harmful, and therefore should be universally targeted. There is evidence that eosinophils also have important developmental roles such as in homeostatic immune protection by augmentation of the longevity of plasma cells,^78^ as well as being activated by viral infections.^79^ However, there is no conclusive evidence in humans that depletion of eosinophils increases viral infections. There is also some evidence that depletion of eosinophils by anti‐IL‐5 antibodies may enhance the antiviral activity of plasmacytoid dendritic cells (pDCs), which may otherwise be suppressed by eosinophils.^80^ Eosinophilic antiviral effects may explain the higher incidence of upper respiratory tract infections in children on dupilumab.^79^, ^81^ In summary, we should remember that eosinophils may have significant physiological roles in children, as well as being the effector cells of Th‐2 inflammation, and complete obliteration (in the case of benralizumab^54^) may not be the ideal solution in the management of severe asthma.

Although we have discussed the evidence of each of the biologics licenced for use in children, there are two further factors worth considering here: (i) omalizumab dosing is calculated based on weight, whereas the other three biologic doses are calculated by age. This may have a larger influence in the efficacy of each biologic in children, and therefore further dosing trials need to be done in children to establish a weight adjusted dosing regime, which may achieve a better therapeutic response in children. (ii) Similar to blood eosinophil levels in adults being extrapolated to decide cut offs for mepolizumab and dupilumab eligibility in children, adult studies were also used to decide IgE ranges for children eligible for omalizumab. However, we know that at least one‐third of children with severe asthma have a higher serum IgE, but efficacy in this group pf children have never been tested. Therefore, further trials need to be carried out in appropriate populations to establish the optimal biomarker cut‐offs in children, if these are to be used as eligibility criteria.^77^

Ultimately, it is essential that the right biologic is prescribed for the right child. Children with STRA must be treated under the supervision of a severe asthma specialist center, so that they have access to an MDT service to optimize treatment before biologics are considered.

## 10 FUTURE DIRECTIONS

Aside from some specific rare reactions, the most common side effects for all four biologics include pyrexia, headaches, soreness at injection sites, abdominal pain, sore throat and sinus pain. With such a good safety profile, it raises the question of why the current guidelines necessitate that doctors should wait for 3–4 attacks to occur within a year before biologics are considered, when it is known that the biggest risk factor for an asthma attack is a previous attack.^82^ If after an asthma attack a second one follows, despite best efforts to optimize basic management by standard means, then perhaps a new approach with a biological is firmly indicated.

With the present state of knowledge, choosing the right biologic for each child is constrained by the limited availability of suitable predictive biomarkers in children and a lack of head‐to‐head studies comparing different biologics in children with severe uncontrolled asthma. Current areas most in need of further research include (1) comparison studies between available biologics, especially in terms of efficacy in patients under 12 years old, (2) identifying stable noninvasive biomarkers that determine the appropriate cohort for each treatment, and which can be measured longitudinally to assess success of treatment, and (3) identifying suitable end points for treatment, and how to maintain control following successful treatment, and when (if ever) treatment can be weaned.

Is there a role for the use of biologics in severe preschool wheeze, in children under the age of 6 years old? The emergence of new data shows that only approximately a quarter of preschool children who wheeze, have the atopic, eosinophilic, Type 2 phenotype,^83^ and this cohort (1) show a better response to inhaled corticosteroids,^84^ which is currently the first line of treatment according to the GINA guideline ^85^ and (2) are at risk of progressing to school aged asthma.^86^, ^87^, ^88^ The PARK study, which is currently ongoing, is a double‐blinded, randomized controlled trial of omalizumab in preschool children who have been deemed high‐risk for developing asthma, and this will provide invaluable evidence as to the role of novel biologics as a secondary preventative therapy, in an attempt to halt subsequent progression to school aged asthma.^89^

We acknowledge that availability of biologics for severe asthma is limited to high‐income countries at present, with omalizumab being the most accessible monoclonal antibody, worldwide.^90^ The high cost of these biologics remains the main constraint. Even in high‐income countries where healthcare is accessed by insurance, its availability is not universal. It should also be noted that the nature of asthma, including the prevalence of non‐eosinophilic asthma, varies across the world.^91^ Undoubtedly a small number of LMIC asthmatics will benefit from biologicals. Omalizumab is coming off patent in November 2025, and there is a large market developing biosimilars. This is likely to become much more accessible worldwide, and given it is still effective in approximately half‐two‐thirds of children, this will be a step forward in LMIC. However, it is crucial that the basic work‐up and management of severe asthma is first addressed. Also important is making the WHO list of essential asthma medications universally available, as targeting public heath interventions to improve disease awareness and address modifiable risk factors in low‐ and middle‐income countries, to to have the greatest impact in improving disease burden.

## AUTHOR CONTRIBUTIONS

KH‐ Writing‐ original draft, literature review. SS‐ Writing‐ reviewing and editing, visualization and supervision. AB‐ Conceptualization, visualization, literature review, writing‐ reviewing and editing.

## CONFLICT OF INTEREST STATEMENT

The authors declare no conflicts of interest.

## Data Availability

Data sharing is not applicable to this article as no new data were created or analyzed in this study.
